# Identification of a Domain which Affects Kinetics and Antagonistic Potency of Clozapine at 5-HT_3_ Receptors

**DOI:** 10.1371/journal.pone.0006715

**Published:** 2009-08-21

**Authors:** Gerhard Rammes, Christine Hosp, Brigitte Eisensamer, Sascha Tanasic, Caroline Nothdurfter, Walter Zieglgänsberger, Rainer Rupprecht

**Affiliations:** 1 Max Planck Institute of Psychiatry, Munich, Germany; 2 Department of Anesthesiology, Technische Universität München, Munich, Germany; 3 Department of Psychiatry and Psychotherapy, Ludwig Maximilians University of Munich, Munich, Germany; Vrije Universiteit Amsterdam, Netherlands

## Abstract

The widely used atypical antipsychotic clozapine is a potent competitive antagonist at 5-HT_3_ receptors which may contribute to its unique psychopharmacological profile. Clozapine binds to 5-HT_3_ receptors of various species. However, the structural requirements of the respective binding site for clozapine remain to be determined. Differences in the primary sequences within the 5-HT_3A_ receptor gene in schizophrenic patients may result in an alteration of the antipsychotic potency and/or the side effect profile of clozapine. To determine these structural requirements we constructed chimeras with different 5-HT_3A_ receptor sequences of murine and human origin and expressed these mutants in human embryonic kidney (HEK) 293 cells. Clozapine antagonises recombinant mouse 5-HT_3A_ receptors with higher potency compared to recombinant human 5-HT_3A_ receptors. 5-HT activation curves and clozapine inhibition curves yielded the parameters EC_50_ and IC_50_ for all receptors tested in the range of 0.6–2.7 µM and 1.5–83.3 nM, respectively. The use of the Cheng-Prusoff equation to calculate the dissociation constant K_b_ values for clozapine revealed that an extracellular sequence (length 86 aa) close to the transmembrane domain M1 strongly determines the binding affinity of clozapine. K_b_ values of clozapine were significantly lower (0.3–1.1 nM) for receptors containing the murine sequence and higher when compared with receptors containing the respective human sequence (5.8–13.4 nM). Thus, individual differences in the primary sequence of 5-HT_3_ receptors may be crucial for the antipsychotic potency and/or the side effect profile of clozapine.

## Introduction

Schizophrenia is a severe psychiatric illness with hallucinations, delusions, poverty of thought and emotions, social withdrawal and cognitive deficits as leading symptoms. A dysregulation of the dopaminergic neurotransmitter system plays an important role in the pathophysiology of schizophrenia. However, current research indicates additional dysfunctions of glutamatergic, GABAergic and also serotonergic (5-HT) neurotransmission [1], [2]. Most antipsychotic agents antagonise the actions of endogenous dopamine at type 2 dopamine (D_2_) receptors in the brain. In contrast, the widely used atypical antipsychotic clozapine has a relatively poor affinity to D_2_ receptors, but exerts also antagonistic effects at histamine receptors, muscarinic acetylcholine receptors, α-adrenoceptors and serotonin receptors [3], [4]. Within the 5-HT receptor subtypes clozapine is a potent antagonist at 5-HT_2_, 5-HT_3A_, 5-HT_6_ and 5-HT_7_ receptors [5].

The dopamine hypothesis of schizophrenia suggests an enhanced mesolimbic activity of dopaminergic neurotransmission [6], [7]. Behavioural, neurochemical and electrophysiological investigations indicate that 5-HT_3_ receptors modulate dopaminergic activity in mesolimbic and nigrostriatal pathways [8], [9]. 5-HT_3_ receptor activation enhanced dopamine release from slices of rat nucleus accumbens [10], striatum [11], [12], and increased the activity of dopaminergic neurons in the ventral tegmental area [13]. These data suggest that 5-HT_3_ receptor antagonists could mimic certain inhibitory effects of antipsychotic drugs. It may therefore be assumed that the antagonistic effects of clozapine mediated via 5-HT_3_ receptors might contribute to its antipsychotic potential.

Functional 5-HT_3_ receptors can only be formed by 5-HT_3A_ subunits, alone or in combination with the 5-HT_3B_ subunit [14]. The functional antagonism of antipsychotics at the 5-HT_3A_ receptor may have important physiological implications. In the CNS, the functional properties of presynaptic 5-HT_3A_ receptors may differ from those of postsynaptic 5-HT_3A_ receptors. Presynaptic 5-HT_3A_ receptors are responsible for the elevation of intracellular Ca^2+^ and modulate the release of several neurotransmitters such as glutamate, dopamine, GABA, norepinephrine and 5-HT [15], [16], [17]. Postsynaptic 5-HT_3A_ receptors mediate fast synaptic neurotransmission in the CNS [18], [19]. The reduction of these Na^+^ and Ca^2+^ fluxes by antipsychotics may be involved in their inhibitory effect on neuronal discharge activity, and modulation of postsynaptic 5-HT_3A_ receptors could alter learning and memory processes [17], [20], [21], [22].

The primary amino acid sequence of the receptor determines the affinity of agonists or antagonists for the specific binding site. The effects of the competitive 5-HT_3A_ receptor antagonist clozapine are affected by either changes in the primary sequences of the 5-HT_3A_ receptor gene encoding for the binding site or by modulation of the binding affinity of the endogenous agonist 5-HT to the receptor. It is therefore possible that variations in the 5-HT_3_ receptor gene of schizophrenic patients may result in an alteration of the antipsychotic potency and/or the side effect profile of clozapine.

Functional antagonistic properties of the atypical antipsychotic clozapine have previously been reported for recombinant mouse 5-HT_3A_ receptors with even higher potency (IC_50_ = 10 nM; [23], [24]) compared to recombinant human 5-HT_3A_ receptors (IC_50_ = 680 nM; [5]). To investigate the structural domains involved in the ligand recognition site for clozapine and activation and deactivation kinetics of 5-HT_3A_ receptors we constructed 5 different receptor chimeras consisting of different murine and human sequences. The antagonistic effects of clozapine and those of 5-HT on receptor kinetics were tested by monitoring cation currents through these different functional receptor mutants.

## Materials and Methods

### Cell culture

Native human embryonic kidney cells (HEK 293 cells) were purchased (German collection of cell cultures, Braunschweig, Germany) and HEK 293 cells stably expressing the human 5-HT_3A_ receptor [25] or the murine 5-HT_3A_ receptor, respectively, were grown as previously described [5].

### Transfection

cDNAs encoding the human 5-HT_3A_ subunit (nucleotides 217–1663, GenBank accession no. D49394, and chimeras were cloned into pCDM8 plasmid vectors [14]), the murine 5-HT_3A_ subunit was cloned into a pCDM6xl plasmid vector. HEK 293 cells were stably transfected with plasmids containing cDNA for the human 5-HT_3A_ or with cDNAs for the murine 5-HT_3A_ subunits_._ Chimeric 5-HT_3A_ receptor subunits or the P391R mutant carrying an intracellular mutation [26] were transiently transfected. A plasmid (pCDM8, pRK5) encoding for the cDNA of green fluorescent protein (GFP) as an expression marker was co-transfected. Exponentially growing HEK 293 cells (2×10^6^ cells) were transfected with chimeric or P391R DNA and GFP DNA by electroporation (BTX Electroporation System, Electro Cell Manipulator 600, San Diego, CA). Cells were harvested 12–18 h before transfection. After harvesting from a 20×100 mm culture dish, the cells were resuspended in an electroporation buffer (975 µl, distilled H_2_0 containing (in mM) 50 K_2_HPO_4_, 20 K^+^-acetate, pH 7.35) and a magnesium-sulfate solution (25 µl distilled water containing 1 M MgSO_4_, pH 6,7) before transfection plasmids containing cDNAs for the 5-HT_3A_ receptor subunits (5 µg) and for GFP (3 µg) were added to the cell suspension. Electroporation was performed at 300 V and 1 mF with a pulse time of 30–45 ms. Transfected cells were replaced in 10×35 mm culture dishes with supplemented medium and incubated (5% CO_2_, 95% air, and 100% relative humidity, 37°C) for 12–18 h before the experiments. After the incubation period, 5–30% of the transfected cells expressed GFP, which is soluble in the cytoplasma, and more than 50% of the green fluorescent cells yielded 5-HT-induced inward currents. The kinetics of 5-HT_3_ receptor-mediated currents in HEK 293 cells with cotransfected GFP were identical to those in preparations without GFP cDNA co-transfection.

### Construction of unique restriction sites in the human and mouse 5-HT_3_ receptor

To create unique restriction sites we introduced a BstEII site in the human 5-HT_3_ receptor subunit [27], corresponding to the BstEII site in the mouse 5-HT_3_ receptor mRNA at position 531 [28]. Additionally, in the murine 5-HT_3_ receptor subunit a SgrA1 restriction site was introduced corresponding to the SgrA1 site in the human 5-HT_3_ receptor mRNA at position 935 and an XhoI multicloning site was introduced in the mouse 5-HT_3_ receptor at position 1541 corresponding to the XhoI multicloning site in the human gene. Mutations were performed with the QuikChange Site-directed Mutagenesis Kit (Stratagene, USA). All mutations were silent.

### Construction of chimeric receptors

To construct chimeric receptors the cDNAs of both the human and the murine receptor subunits were digested with BstEII and HindIII, SgrA1 and HindIII, SgrA1 and BstEII, SgrA1 and XhoI, BstEII and XhoI, respectively. The digestion products were resolved on 1% or 1.5% agarose gels. The resulting small murine digestion fragments were subcloned in the corresponding human vector fragments. The chimeric cDNAs were sequenced on both strands to verify integrity of the mutants.

### Structure of 5-HT_3_ receptor chimeras composed of human and murine 5-HT_3A_ sequences

**Figure 1:**
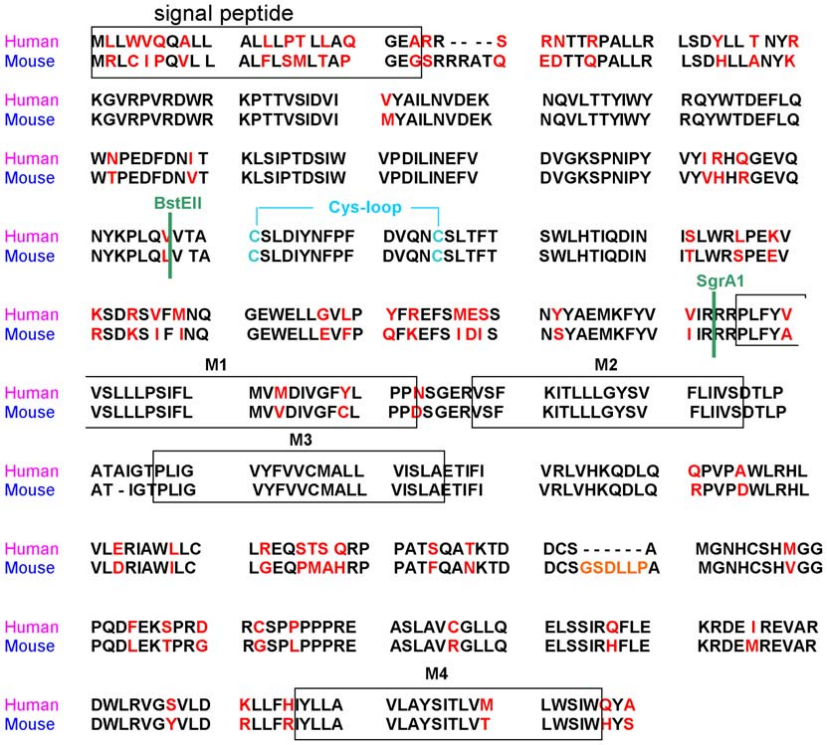
Amino acid sequence of cloned cDNA encoding the human and mouse 5-HT_3A_ receptor channel subunit. Marked in red: mismatches of the amino acid sequence. Marked in green: Restriction sites for BstEII and SgrA1 representing switching points of the chimeric receptors. C-C: Cys-loop. M1–M4 transmembrane segments.

**Figure 2:**
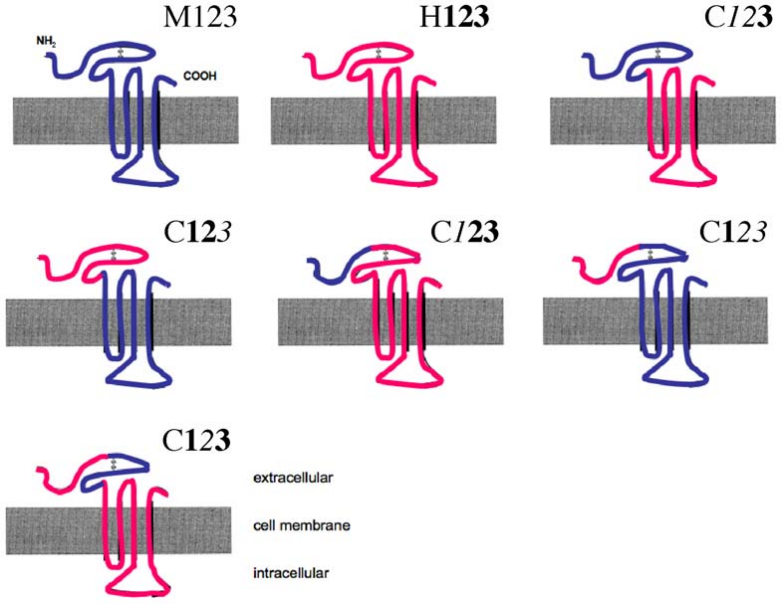
Different receptor chimeras and the respective sequences consisting of human and mouse 5-HT_3A_ receptor composites. For clarity, human, murine receptors and chimeras are indicated as a combination of the numbers for the three different sequences, where human sequences are marked in bold and murine sequences are marked in italics. The prefix “H”, “M” and “C” indicates human, murine and chimeric receptors, respectively: human 5-HT_3_ receptor = H123, murine 5-HT_3_ receptor = M*123*, chimeric receptors are C1*23*, C12*3*, SH1,C*12*3, C*1*23 and C1*2*3.

5-HT_3A_ receptors are pentameric assemblies of subunits consisting of extracellular, transmembrane, and cytoplasmic domains [29]. To investigate the molecular determinants for the differences in receptor kinetics, affinity and antagonistic potency of clozapine we constructed different chimeric receptors between human and murine 5-HT_3_ receptor sequences. We created five chimeric receptors (Fig. 1 and 2), which contained the sequence between the amino terminus ( = restriction site HindIII) and restriction site of BstEII (defined as sequence 1) and the sequence between restriction site BstEII and SgrA1 (defined as sequence 2). Sequence 1 and 2 together belong to the extracellular domain and form the ligand binding site. The sequence between restriction site of SgrA1 and the carboxyl-terminal domain ( = restriction site XhoI; defined as sequence 3; Fig. 2) belongs to the transmembrane and cytoplasmic domain. For clarity, human, murine receptors and chimeras were indicated as a combination of the numbers for the three different sequences where human sequences are marked in bold and murine sequences are marked in italics. The prefix “H”, “M” and “C” indicates human, murine and chimeric receptors, respectively: human 5-HT_3_ receptor = H**123**, murine 5-HT_3_ receptor = M*123*, chimeras are either C**1** *23*, C**12** *3*, C*12* **3**, C*1* **23** or C**1** *2* **3**. Fig. 1A shows the different receptor chimeras and the respective sequences consisting of human and mouse 5-HT_3_ receptor composites. The expression plasmid carrying the P391R mutant [26] was kindly provided by Sarah Lummis, Cambridge, UK.

### Concentration clamp recordings

5-HT-induced inward Na^+^ currents were recorded from lifted HEK cells transiently transfected with the human, murine 5-HT_3A_ receptor and chimeras in the whole-cell voltage clamp configuration under visual control using an inverted microscope (Zeiss, Jena, Germany) as previously described [30]. Cells were kept in a bath solution containing 140 mM NaCl, 2.8 mM KCl, and 10 mM HEPES, pH 7.2. Patch electrodes were pulled from borosilicate glass (Hilgenberg, Malsfeld, Germany) using a horizontal pipette puller (Zeitz Instruments, Augsburg, Germany) to yield pipettes with a resistance of 3–6 MΩ. Pipettes were filled with a solution containing 130 mM CsCl, 2 mM MgCl_2_, 2 mM CaCl_2_, 2 mM ATP, 0.2 mM Tris-GTP, 10 mM glucose, 10 mM HEPES, and 10 mM EGTA, pH 7.2. After the whole-cell configuration was established, the cells were lifted from the glass substrate and 10 µM 5-HT were applied using a fast superfusion device. We applied these concentrations since 10 µM 5-HT were used for the determination of the IC_50_ value for the inhibition of the 5-HT response by psychopharmacological drugs in our previous study [5], which was in the low micromolar range. For control experiments a piezo translator-driven double-barrelled application pipette was used to expose the lifted cell either to 5-HT-free or 5-HT-containing solution. A 2 s 5-HT pulse was delivered every 90 s.The stock solutions (10 mM or 10 µM) of clozapine were diluted with bath solution to the desired concentration. To control for any possible confounding solvent effects, currents were recorded with 0.1% ethanol in 5-HT-free or 5-HT-containing solutions. Current signals were recorded at a holding potential of −50 mV with an EPC-9 amplifier (Heka, Lamprecht, Germany) and were analysed using the Heka 8.5 PulseFit and IgorPro v. 5.04B (Wavemetrics, Lake Oswego, OR, USA) software on a Power Macintosh G3 computer. In experiments with clozapine, only results from stable cells entered the final analysis, that is, showing at least 50% recovery of responses to 5-HT following the removal of drugs. In some cells, recovery was not 100% because of rundown (see frequent activation experiments). To compensate for this effect the % antagonism at each concentration was based on both the control and the recovery current by assuming a linear time course for the rundown. Data are shown as mean ±SEM. We recorded from a total of 120 cells. Measurements were performed as independent experiments relative to control and recovery.

Dose-response curves and the respective EC_50_ and IC_50_ values were calculated by the four parameter logistic equation for agonists: I = I_max_ (agonist)^Hill^/(agonist+EC_50_^Hill^) and for antagonists: I = I_max_ (antagonist)^Hill^/(antagonist+IC_50_^Hill^). The corrected binding affinity of clozapine K_b_ was calculated using the Cheng-Prusoff equation [31]: K_b_ = IC_50_/(1+[agonist]/EC_50_). A full dose-response curve was determined from every cell and the EC_50_ and IC_50_ values with the respective Hill coefficients were calculated. These values from each single dose-response curve were averaged thereafter. Thus, the reported means ±SEM result from different cells after averaging. For the figures of the dose-response curves we fitted the curve according to the average value for each respective concentration. Because the charge represents the most appropriate measure for receptor activation the IC_50_ and EC_50_ values for charge entered the Cheng-Prusoff equation for K_b_ analysis.

### Statistical analysis

EC_50_ and IC_50_ values, respectively, were calculated for each recorded cell and tested for statistical significance by one-way ANOVA using Bonferroni correction for multiple comparison. For testing the rundown of the different 5-HT_3_ receptors after multiple activations, the values of the last 5 5-HT applications (22^nd^–26^th^ application) entered statistical analysis. A value of *p*<0.05 was considered as statistically significant. Statistical analysis was performed using the SPSS 14.0 for Windows (SPSS Inc., Chicago, IL, USA).

### Chemicals and Drugs

Clozapine was purchased from Sigma (Munich, Germany) and stock solutions (10 mM) were prepared in pure ethanol. Thus, the maximum ethanol concentration in experiments using drug concentrations of 10 µM was less than 0.1%. Serotonin was purchased from Sigma and dissolved in water.

## Results

### Rundown kinetics of recombinant human and murine 5-HT_3_ receptors after multiple activation

**Figure 3:**
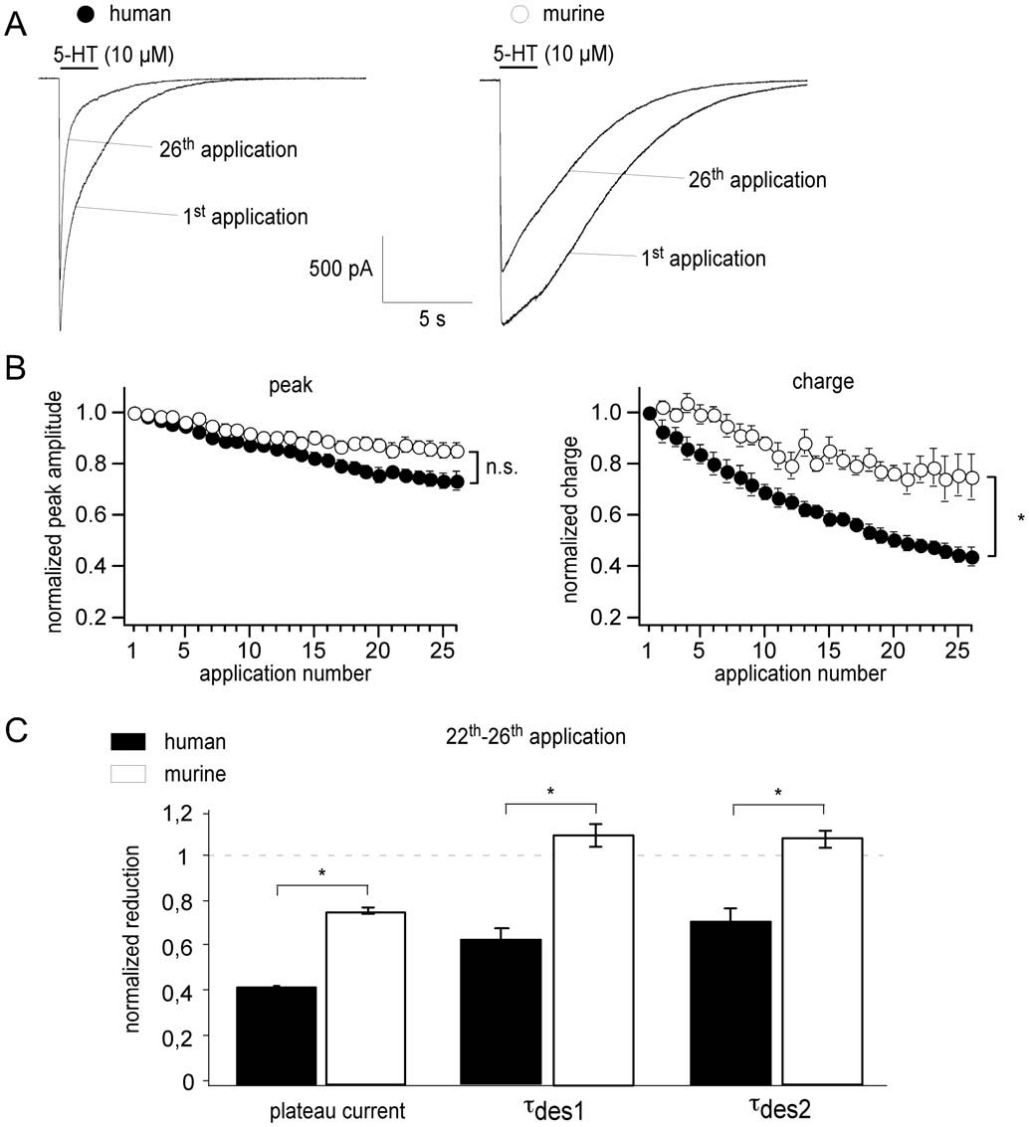
Rundown kinetics of recombinant human and murine 5-HT_3A_ receptors after multiple activation. (A) Representative current traces for human (left) and murine (right) receptors showing currents after the first and 26^th^ application of 5-HT. Records were obtained from the same cell. (B) Repeated 5-HT applications (26 applications within 40 min) reduced peak currents (left) and charge (right) through H123 (filled circles) and M*123* (open circles) differently. Since rundown affects plateau currents and desensitization of H123 more effectively than those of M*123*, the calculation of charge thus represents a very sensitive parameter for receptor activity. (C) Bar diagram showing the change in plateau currents, t_des1_ and t_des2_ after 22 to 26 5-HT applications (mean ± SEM). Values were normalized to current kinetics evoked by the first application of 5-HT.

5-HT_3_ receptor activity is very sensitive to the presence of external Ca^2+^ as currents decline when Ca^2+^ concentration increases [32]. Therefore, 5-HT-activated currents were recorded in a HEPES-buffered Ca^2+^-free solution. First, we characterized the kinetics of H**123** and M*123* (see also Table 1). At a holding potential of −50 mV, 5-HT (10 µM) applied for 2 s evoked inward currents which rose (H**123**: t_on_ = 20.1±1.5 ms, n = 38; M*123*: t_on_ = 28.5±1.6 ms, n = 36; Fig. 3A) to a peak of 3806.1±272.5 pA and 340.3±30.3 pA for H**123** and M*123*, respectively, and induced an incomplete receptor desensitization (H**123**: t_des1_ = 1879.7±164.3 ms, t_des2_ = 1359.4±161.7 ms; M*123*:t_des1_ = 2647.6±132.9 ms, t_des2_ = 2514.8±148.3 ms; Fig. 3A). Because of these characteristics of receptor desensitization, true steady-state responses could not be determined. Hence, steady-state currents have been defined as the last 10 ms of 5-HT application (see also [5], [25]). After the removal of 5-HT, receptor currents deactivated completely with a time constant of t_off_ = 1977.0±117.7 ms and t_off_ = 5937.4±730.1 ms for H**123** and M*123*, respectively (Fig. 3A).

**Table 1:** Kinetics of human, murine and chimeric 5-HT_3A_ receptors after first application of 5-HT for 2 s.

	Peak (pA)	Charge (pC)	t_on_ (ms)	t_des1_ (ms)	t_des1_ (ms)	t_off_ (ms)	plateau (pA)
C*1* **23**	−1045.8	−4.49	18.3	2053.7	1599.4	3951.5	−684.3
	±210.9	±1.27	±2.9	±149.7	±198.1	±234.5	±171.5
C**12** *3*	−886.7	−3.01	59.7	2190.1	1726.5	2355.9	−534.6
	±142.2	±0.8	±3.1	±232.7	±274.6	±184.1	±122.5
C*12* **3**	−2446.1	−10.6	8.7	2266.8	1580.8	3459.6	−1628.8
	±252.5	±1.51	±0.6	±161.9	±212.0	±254.0	±230.9
C**1** *2* **3**	−2003.4	−7.5	10.7	2310.6	1456.2	2272.6	−1430.2
	±193.7	±0.9	±1.6	±141.1	±246.5	±628.3	±158.6
C**1** *23*	−1667.1	−10.9	39.5	2384.4	2131.8	2910.3	−1517.9
	±256.8	±1.9	±5.3	±179.6	±199.7	±302.2	±249.9
M*123*	−340.3	−2.1	28.5	2647.6	2514.8	5937.4	−294.3
	±30.3	±0.3	±1.6	±132.9	±148.2	±730.1	±31.7
H**123**	−3806.1	−10.1	20.1	1879.7	1359.4	1977.0	−2047.4
	±272.5	±1.2	±1.5	±164.3	±161.7	±117.7	±235.0

The frequent activation of human 5-HT_3_ receptors is accompanied by constant rundown kinetics as previously shown [5]. In the present study, multiple 5-HT applications (26 applications within 40 min) reduced peak currents through H**123** to 74% (n = 4), accelerated t_des1_ and t_des2_ to 64% and 73%, respectively, and reduced the steady-state current to 37% (Fig. 3B, C).

Rundown kinetics of M*123* are significantly less pronounced (after 26 applications, peak currents were reduced to 84%, plateau currents to 76% and t_des1_ and t_des2_ were even slowed down to 110% and 109%, respectively; Fig. 3B, C). Since rundown affects plateau currents and desensitization of H**123** more effectively than those of M*123*, the calculation of charge thus represents a very sensitive parameter for receptor activity. Fig. 3B (right) demonstrates the strong rundown of H**123** charge to 42% of control in comparison to the reduction of M*123* charge to 72%. The parameter charge is therefore a suitable tool to investigate the molecular determinants for the differences in receptor kinetics by constructing human/murine receptor chimeras.

In the present study, we found a 99.4% recovery for human and a 97.9% recovery for murine receptors of the peak amplitude when two 5-HT applications for 2 sec were separated by a 25 sec interval (data not shown). These results are consistent with previous reports [33], [34] which found a nearly complete recovery after 25 to 60 sec. Thus, using an interval of 90 sec between two 5-HT applications as in the present study should not affect receptor desensitization and prevent consecutive accumulation of desensitization. Furthermore, a similar rundown was obtained for both human and murine 5-HT_3_ receptors after only two 5-HT applications separated by a 40 min interval (data not shown). As such, the rundown cannot be simply attributed to an enhancement of receptor desensitization induced by multiple 5-HT applications.

### Rundown kinetics for chimeras

**Figure 4:**
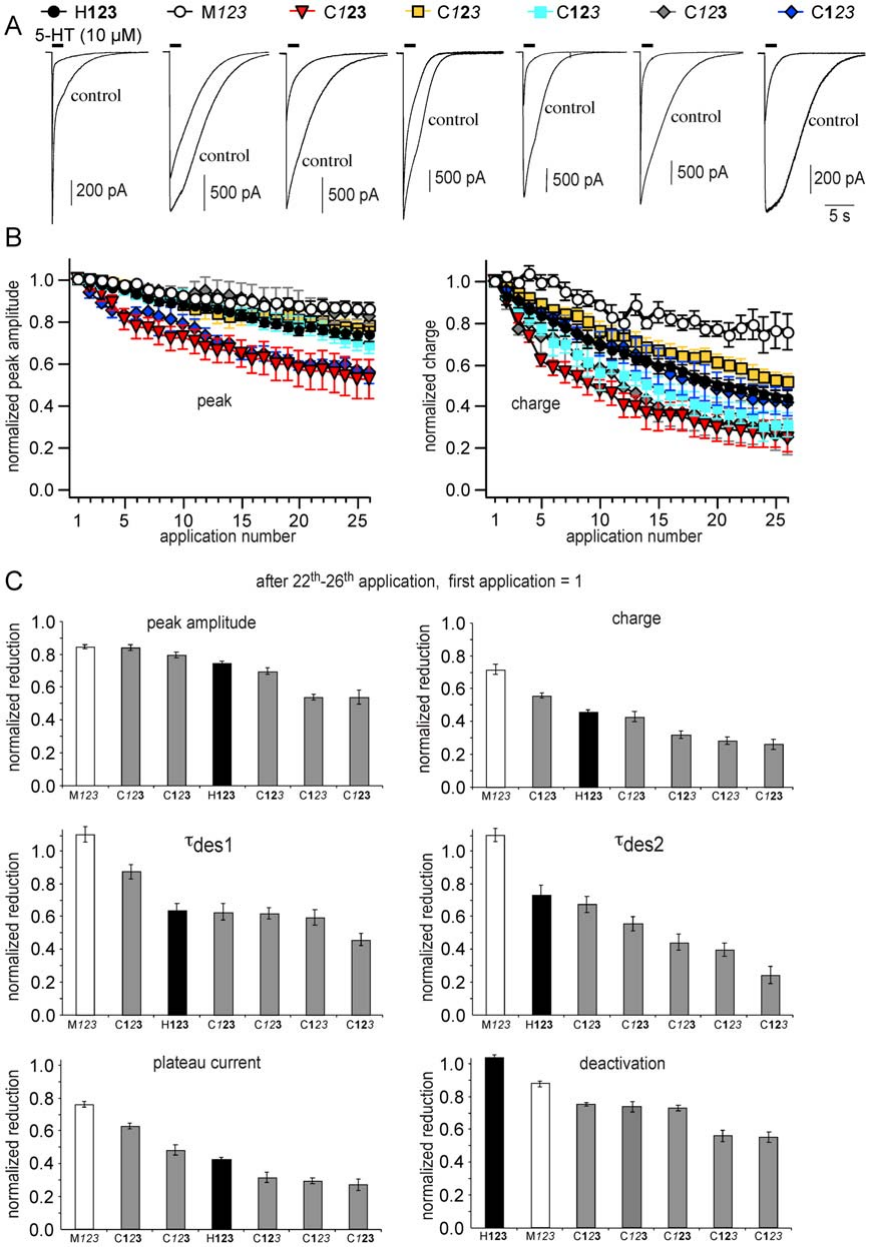
(A) Rundown kinetics for chimeras. Representative current traces for human, murine receptors and chimeras showing currents after the first and 26^th^ application of 5-HT. Records were obtained from the same cell. All chimeras tested produced functional currents upon 5-HT activation. However, the currents through the different receptor chimeras showed a strong variation in kinetics and rundown. (B) Repeated 5-HT-applications (26 applications within 40 min) reduced peak currents (left) and charge (right). Differences were only marginally for peak currents, whereas analysis of charge showed more pronounced variation between all receptor types. (C) Charge variations between the different receptor types displayed a good correlation to kinetic parameters such as desensitization, plateau current and deactivation.

All chimeras tested produced functional currents upon 5-HT activation. However, the currents through the different receptor chimeras showed a strong variation in kinetics (Table 1) and rundown (Fig. 4A, B, C ; Table 2, 3). Whereas these differences were only marginally for peak currents (except for C**1** *23* and C*1* **23** where amplitudes were significantly reduced to 58% and 58% of control, respectively), analysis of charge showed more pronounced variation between all receptor types (Fig. 4B, C). Concomitantly, charge variations between the different receptor types displayed a good correlation to kinetic parameters such as desensitization, plateau current and deactivation (Fig. 4C). The M*123* receptor currents were less affected by multiple 5-HT applications, showing only a minor rundown. Statistical analysis revealed that the charge of all other receptor types was strongly reduced in comparison to M*123*. No significant difference could be found between either C**1** *2* **3**, H**123**, C*12* **3** and C**12** *3*, C**1** *23*, C*1* **23**, respectively. However, each of the receptor types C**12** *3*, C**1** *23* and C*1* **23** showed a significantly reduced charge compared to C**1**2**3**, H**123** and C12**3**.

**Table 2:** Differences in rundown with regard to peak amplitude after multiple activation for human, murine and chimeric receptors.

receptor	M*123*	C*12*3	C1*2*3	H123	C12*3*	C*1*23	C1*23*
M*123*							
C*12* **3**							
C**1** *2* **3**							
H**123**							
C**12** *3*	•	•	•				
C*1* **23**	•	•	•	•	•		
C**1** *23*	•	•	•	•	•		

Significant differences (p<0.05, ANOVA) are indicated by black circles.

**Table 3:** Differences in rundown with regard to charge after multiple activation for human, murine and chimeric receptors.

receptor	M*123*	C1*2*3	H123	C*12*3	C12*3*	C*1*23	C1*23*
M*123*							
C**1** *2* **3**	•						
H**123**	•						
C*12* **3**	•	•					
C**12** *3*	•	•	•	•			
C*1* **23**	•	•	•	•			
C**1** *23*	•	•	•	•			

Significant differences (p<0.05, ANOVA) are indicated by black dots.

### Functional antagonistic properties of the atypical antipsychotic clozapine against H123 and M*123* currents

**Figure 5:**
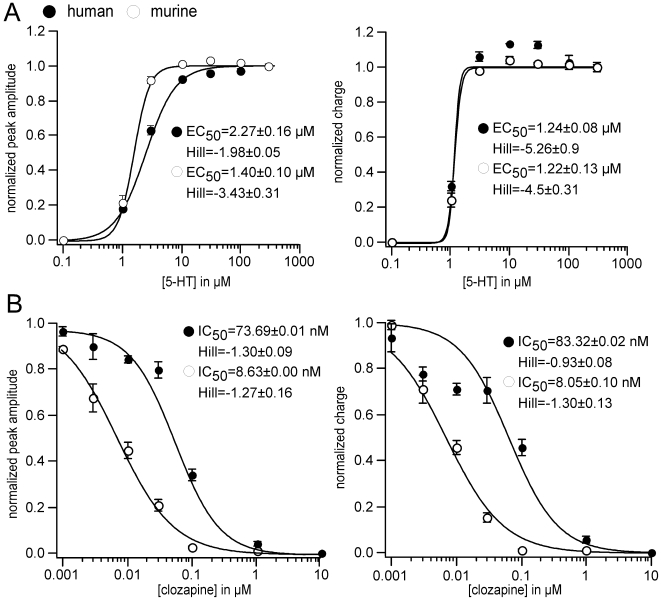
5-HT affinity and functional antagonistic properties of the atypical antipsychotic clozapine against H123 and M*123* currents. (A) Human and murine 5-HT_3_ receptors showed almost identical affinity to 5-HT. (B) Clozapine antagonised 5-HT activated currents through human and murine 5-HT_3_ receptors with different potencies, allowing the identification of the structural domains involved in the ligand recognition for clozapine by human/murine chimeras.

Human and murine 5-HT_3_ receptors showed almost identical affinity to 5-HT [25] which was also confirmed in the present study. Peak amplitude and charge of currents through H**123** and M*123* were concentration-dependently increased (Fig. 5A). Clozapine antagonised 5-HT-activated currents through human and murine 5-HT_3_ receptors with different potencies [5], [24]. In the present study, clozapine was significantly more potent against M*123* whereas the peak amplitude and charge of H**123** were reduced less effectively (Fig. 5B). As such, the structural domains involved in the ligand recognition for clozapine can be identified by human/murine chimeras.

### 5-HT-induced currents through chimeric receptors

**Figure 6:**
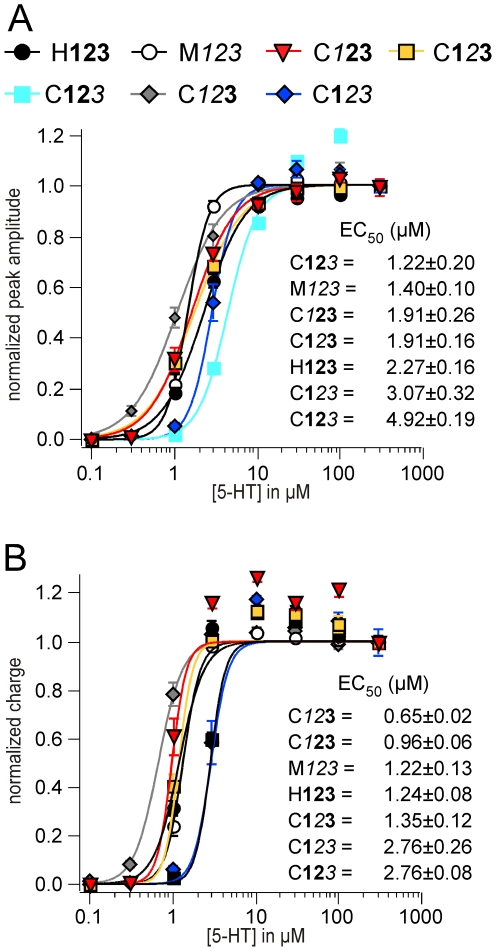
(A, B) 5-HT-induced currents through chimeric receptors. All chimeric receptors were dose-dependently activated by 5-HT. For each dose-response curve values were normalized to the responses induced by 300 µM 5-HT. Dose-response curves for amplitude (A) and charge (B).

All chimeric receptors were dose-dependently activated by 5-HT with an EC_50_ in the range of 1.22 µM to 4.92 µM and 0.65 µM to 2.76 µM for peak and charge, respectively (Fig. 6A, B; Table 4, 5). For each dose-response curve values were normalized to the responses induced by 300 µM 5-HT. The EC_50_ for the peak current through the chimera C**12** *3* significantly differed from all other receptor types, that from C**1** *23* only from M*123* and C*12* **3**. The EC_50_ for the peak current for C*12* **3**, M*123*, C*1* **23**, C**1** *2* **3** and H**123** were comparable with regard to the EC_50_ for charge: C**1** *23* and C**12** *3* showed the lowest affinity for 5-HT with an equal EC_50_ which differed significantly from those of all other receptor types. The dose-response curves for 5-HT of C*12* **3**, C*1* **23**, M*123*, H**123** and C**1** *2* **3** yielded a similar EC_50_ with no significant difference (Fig. 6 A, B).

**Table 4:** Potency of serotonin for human, mouse and chimeric 5-HT_3A_ receptors against peak amplitude.

receptor	C*12*3	M*123*	C*1*23	C1*2*3	H123	C1*23*	C12*3*
C*12* **3**							
M*123*							
C*1* **23**							
C**1** *2* **3**							
H**123**							
C**1** *23*	•	•					
C**12** *3*	•	•	•	•	•	•	

Significant differences (p<0.05, ANOVA) are indicated by black dots. Comparisons of EC_50_ for peak amplitude.

**Table 5:** Potency of serotonin for human, mouse and chimeric 5-HT_3A_ receptors against charge.

receptor	C*12*3	C*1*23	M*123*	H123	C1*2*3	C1*23*	C12*3*
C*12* **3**							
C*1* **23**							
M*123*							
H**123**							
C**1** *2* **3**							
C**1** *23*	•	•	•	•	•		
C**12** *3*	•	•	•	•	•		

Significant differences (p<0.05, ANOVA) are indicated by black dots. Comparisons of EC_50_ for charge (B).

### Antagonistic properties of clozapine against chimeric and mutant receptors

**Figure 7:**
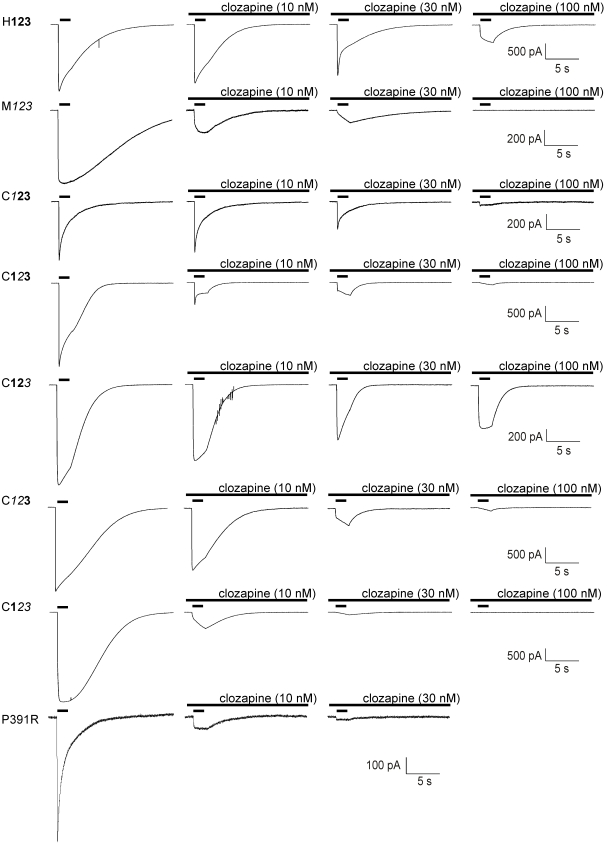
Antagonistic properties of clozapine against chimeric and mutant receptors. All 5-HT-induced currents through chimeric receptors and the P391R mutant were dose-dependently reduced by clozapine in a competitive manner, however, with different potencies. Representative traces for the control and the effects of different concentrations of clozapine on chimeric 5-HT_3_ receptor-mediated currents. The application duration of 5-HT was 2 s.

**Figure 8:**
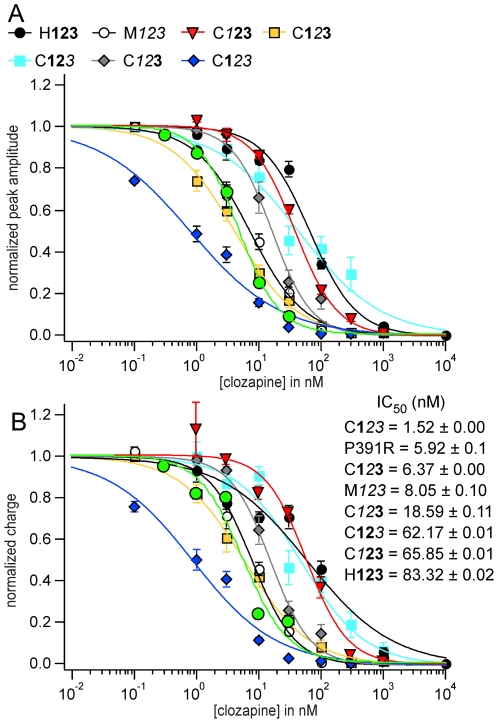
(A, B) Antagonistic properties of clozapine against chimeric and mutant receptors. All 5-HT-induced currents through chimeric receptors and the P391R mutant were dose-dependently reduced by clozapine in a competitive manner, however, with different potencies. Dose-response curves for amplitude (A) and charge (B).

All 5-HT-induced currents through chimeric receptors were dose-dependently reduced by clozapine in a competitive manner, however, with different potencies (Fig. 7 and 8; Table 6, 7). Clozapine was most potent against 5-HT-induced currents of C**1** *23* receptors and showed lowest affinity to antagonise currents through H**123** receptors (Fig. 7 and 8). Interestingly, clozapine exerts higher antagonistic potencies against those receptors carrying the murine sequence *2*, whereas clozapine antagonism was less potent against receptors with a corresponding human sequence.

**Table 6:** Potency of clozapine for human and murine 5-HT_3A_ receptors and different 5-HT_3A_ receptor chimeras against peak amplitude.

receptor	C1*23*	C1*2*3	M*123*	C*12*3	C*1*23	C12*3*	H123
C**1** *23*							
C**1** *2* **3**							
M*123*							
C*12* **3**							
C*1* **23**							
C**12** *3*	•	•					
H**123**	•	•	•				

Significant differences (p<0.05, ANOVA) are indicated by black dots. Comparisons of IC_50_ for peak amplitude.

**Table 7:** Potency of clozapine for human and murine 5-HT_3A_ receptors and different 5-HT_3A_ receptor chimeras against charge.

receptor	C1*23*	C1*2*3	M*123*	C*12*3	C12*3*	C*1*23	H123
C**1** *23*							
C**1** *2* **3**							
M*123*							
C*12* **3**							
C**12** *3*							
C*1* **23**							
H**123**	•	•	•				

Significant differences (p<0.05, ANOVA) are indicated by black dots. Comparisons of IC_50_ for peak charge.

Recently, a single point mutation in the cytoplasmic domain of the 5-HT_3_ has been identified in individuals diagnosed with schizophrenia [35]. To investigate whether a mutation located at the intracellular site of the receptor affects competitive antagonism, we tested the pharmacological potency of clozapine against the human P391R mutant [26]. Interestingly, clozapine reduced 5-HT-evoked currents through this transiently transfected receptor with higher potency as for H**123** (IC_50_ for peak: 4.81±0.18 nM (Hill = −1.35); charge: 5.91±0.1 nM (Hill = 1.21); Fig 7 and 8).

### Calculation of the dissociation constant K_b_ of clozapine using the Cheng-Prusoff equation

The agonist activation curves and antagonist inhibition curves in the presence of a fixed agonist concentration [A] have been fitted to logistic functions to yield the parameters EC_50_ and IC_50_. K_b_ for all receptors were obtained with the Cheng-Prusoff estimate (Table 8). The lowest K_b_ for clozapine were calculated for receptors containing the murine sequence 2 (0.3–1.1 nM). When 5-HT_3_ receptors contained the human sequence **2**, clozapine affinity to the binding site was less potent and K_b_ ranged from 5.8–13.4 nM. These data demonstrate that the sequence between restriction site BstEII and SgrA1 (defined as sequence 2) of 5-HT_3_ receptors is important for the binding affinity of clozapine. The calculation of the dissociation constant of clozapine for the P391R mutant revealed a K_b_ of 1.3 nM. The EC_50_ for 5-HT (2.73±0.01 µM) has been taken from Thompson et al. (2006) [26].

**Table 8:** Calculation of the dissociation constant K_b_ of clozapine using the Cheng-Prusoff equation.

receptor	K_b_ clozapine (nM)	EC_50_ 5-HT (µM)	IC_50_ clozapine (nM)
C**1** *23*	0.3	2.76±0.26	1.52±0.00
C**1** *2* **3**	0.8	1.35±0.12	6.37±0.00
M*123*	0.9	1.22±0.13	8.05±0.10
C*12* **3**	1.1	0.65±0.02	18.59±0.11
P391R	1.3	2.73±0.06	5.92±0.1
C*1* **23**	5.8	0.96±0.06	65.85±0.01
H**123**	9.2	1.24±0.08	83.32±0.02
C**12** *3*	13.4	2.76±0.08	62.17±0.01

The lowest K_b_ for clozapine were calculated for receptors containing the murine sequence 2 (0.3–1.1 nM). When 5-HT_3_ receptors contained the human sequence 2, clozapine affinity to the binding site was less potent and K_b_ ranged from 5.8–13.4 nM. These data demonstrate that the sequence between restriction site BstEII and SgrA1 (defined as sequence **2**) of 5-HT_3_ receptors is important for the binding affinity of clozapine. The calculation of the dissociation constant of clozapine for the P391R mutant revealed a K_b_ of 1.3 nM. The EC_50_ for 5-HT (2.73±0.01 µM) has been taken from Thompson et al. (2006) [26].

## Discussion

In the present study we generated five chimeras with different 5-HT_3A_ receptor sequences of murine and human origin to determine the structural basis for clozapine binding. Analysis of 5-HT activation curves and clozapine inhibition curves using the Cheng-Prusoff equation revealed an extracellular sequence (length 86 aa) close to the transmembrane domain M1 which strongly determines the binding affinity of clozapine. These results suggest that genetic variations within this sequence of the 5-HT_3_ receptor gene may contribute to the antipsychotic potency and/or the side effect profile of clozapine under clinical conditions.

There is now good evidence that both homomeric (5-HT_3A_) and heteromeric (5-HT_3AB_) 5-HT_3_ receptor isoforms exist in brain and peripheral neuronal tissue [9], [36]. However, electrophysiological and immunohistochemical evidence indicates that the majority of native 5-HT_3_ receptor complexes do not contain the 5-HT_3B_ subunit [36], [37]; for review see [38]. Moreover, the 5-HT_3B_ subunit does not contribute to the ligand-binding site [39]. Therefore, we focussed only on homomeric 5-HT_3A_ receptors in the present study.

The amino acid sequence of the human 5-HT_3A_ receptor displays 85% identity with the mouse subunits [40]. In contrast to guinea pig receptors, murine and human 5-HT_3_ receptors exhibit a somewhat similar pharmacological profile [25]. The human and the mouse receptor differ only marginally in their affinity to the natural ligand 5-HT [14], [25], [33], [34] which could also be confirmed in the present study. However, both homo-oligomeric receptor types show remarkable differences with regard to receptor kinetics and clozapine affinity. Human 5-HT_3A_ receptors are characterised by a lower charge transfer due to faster desensitization and deactivation kinetics and a more pronounced receptor desensitization (see also [25]). Furthermore, repeated activation of human 5-HT_3A_ receptors produced a marked decline in charge transfer. Currents through mouse receptors are less affected. These differences in rundown kinetics can be best explained by a considerable acceleration of desensitization kinetics and plateau currents for human 5-HT_3A_ receptors, whereas these parameters are only marginally affected in mouse 5-HT_3A_ receptors. Moreover, with regard to pharmacology, the competitive antagonist clozapine more potently inhibits currents through mouse 5-HT_3A_ receptors which is reflected by a 10-fold smaller IC_50_ value than that obtained for human 5-HT_3A_ receptors (see also [5], [24]).

These functional and pharmacological differences may be valuable for locating sequences important for rundown kinetics induced by repeated receptor activation and for clozapine binding affinity by creating human/mouse 5-HT_3A_ receptor chimeras. The extracellular domain, which forms the ligand binding site [32], [41], appears to be crucial for these functions. In the present study we therefore constructed three chimeric receptors (C**1** *2* **3**, C*1* **23**, C**1** *23*) with an extracellular domain combined of human and murine sequences allowing a more detailed mapping of determinants of the agonist and antagonist binding site.

The frequent activation of 5-HT_3A_ receptors is accompanied by constant rundown kinetics reflected by a pronounced acceleration of t_des1_ and t_des2_ and a strong reduction of the steady-state current. Although the peak amplitude also decreased after multiple 5-HT applications, this effect is less prominent. Thus, the charge transfer is the most suitable parameter for assessing receptor activity. Analysis of rundown kinetics revealed a significantly smaller decline in charge transfer for murine receptors compared to human and all chimeric receptors. This decline is predominantly due to a pronounced receptor desensitization after repeated activation, as plateau currents are reduced to a similar degree. Since human and murine receptors recovered almost completely when two 5-HT applications were separated by a 25 sec interval (data not shown), the use of a 90 sec interval should not affect receptor desensitization and should prevent consecutive accumulation of desensitization. Interestingly, a similar rundown is observed for both human and murine 5-HT_3_ receptors after only two 5-HT applications separated by a 40 min interval. Thus, the rundown cannot be simply attributed to an enhancement of receptor desensitization induced by multiple 5-HT applications.

The analysis of the kinetic and rundown characteristics of all chimeras did not unravel a clear sequence correlation. Single activation of C*1* **23** and C**1** *23* produced receptor kinetics similar to murine receptors, whereas repeated activation of these chimeras induced strong rundown kinetics. Conversely, likewise murine receptors, the chimera C**1** *2* **3** showed only a marginal reduction in charge transfer after repeated activation but kinetics similar to human receptors. These results indicate that the molecular determinants responsible for rundown kinetics and for receptor desensitization are not associated with the same protein segment.

5-HT activates human and mouse 5-HT_3A_ receptors with similar potency suggesting that the EC_50_ values for chimeras should not differ significantly. However, when considering the EC_50_ for charge and peak, 5-HT was significantly more potent in activating the chimeras C*1* **23** and C*12* **3** compared to C**1** *23* and C**12** *3*. A plausible explanation for this inconsistency might be that the extracellular domain is not the single determinant for agonist affinity. This hypothesis is supported by a recent investigation demonstrating that a mutation in the cytoplasmic domain (P391R) can also cause alterations in agonist binding [26]. Moreover, irrespective of sequence composition, desensitization and deactivation parameters of chimeric receptors were constantly slower than the fast human receptor kinetics (see table 1). These observations cannot be explained by a simple sequence-to-function correlation. There is evidence that the extracellular domain determines agonist binding [32], [41] whereas the cytoplasmic domain of the 5-HT_3A_ receptor contributes to a receptor desensitization mechanism [25], [42], [43], [44]. Concerning the molecular parameters for agonist affinity, receptor kinetics and rundown, our data rather favour the hypothesis of an involvement of the tertiary and quaternary structure of the whole receptor molecule than a restricted structural domain [32]. It is likely that the successful spatial coupling of the neurotransmitter binding site to the ion channel and cytoplasmic domain is crucial for mediating 5-HT binding, kinetics and rundown properties. This assumption is supported by the fact that a P391R point mutation in the cytoplasmic domain of the 5-HT_3_ receptor affects the agonist binding site by increasing the EC_50_ for 5-HT [35], [26]. Since the rapid desensitization of 5-HT_3A_ receptors during sustained activation [45], [46], [47], [48] has great importance for synaptic regulation [16], alterations of the tertiary and quaternary structure may also have implications in the pathophysiology of schizophrenia.

Clozapine potently antagonises murine [24] and human 5-HT_3A_ receptors [5] with a 10-fold higher affinity against murine receptors. The evaluation of the antagonistic potency of clozapine against chimeric receptors revealed IC_50_ values below the IC_50_ for human receptors and for C**1** *23* and C**1** *2* **3** IC_50_ levels even below those of murine receptors. As clozapine is a competitive antagonist, calculation of true dissociation constant K_b_ values for each receptor needs the consideration of the specific 5-HT affinity. Estimation of K_b_ values for clozapine using the Cheng-Prusoff relationship revealed that sequence 2 of the extracellular ligand binding site (length 86 aa) close to the transmembrane domain M1 strongly determines the binding affinity of clozapine. When chimeric receptors contain the murine sequence 2 (C**1** *23*, C**1** *2* **3**, C*12* **3**), their K_b_ values for clozapine affinity were similar to the K_b_ for murine receptors and significantly lower (0.3–1.1 nM) compared to the chimeras C*1* **23**, C**12** *3* containing the human sequence 2 with K_b_ values of 5.8 and 13.4 nM, respectively.

In contrast to other 5-HT receptors, the HTR_3A_ gene shows a relatively high variability in the coding region, and it is possible that approximately 1% of schizophrenic patients carry 5-HTR_3A_ mutations [35]. Approximately 30–60% of all schizophrenic patients fail to respond to typical antipsychotics [49] and hence, clozapine may be a valuable treatment alternative. The concentrations of clozapine in the cerebrospinal fluid under therapeutical conditions range from 70–130 nM. Genetic variations in the primary sequence of 5-HT_3_ receptors may be crucial for the antipsychotic potency and/or the side effect profile of clozapine in that they may determine the antagonistic properties against this ligand-gated ion channel. Recently, a missense mutation P391R residing in the highly conserved cytoplasmic region has been found, which probably only occurs in schizophrenic patients [35]. The functional characterization of these mutants revealed a significant increase in the EC_50_ for 5-HT of the P391R mutant [26]. Consequently, this mutation may thereby also affect clozapine pharmacology. In fact, the experiments with the P391R mutant revealed a considerable increase in the antagonistic potency of clozapine. Furthermore, the calculation of Kb for clozapine demonstrates that the intracellularly located point mutation affects the extracellular binding for clozapine via two mechanisms: either directly by increasing the binding affinity and/or indirectly by decreasing the EC50 for 5-HT.

In previous studies, two novel 5-HT_3A_ polymorphisms, 178-T/Cand 1596-A/G, have been reported [50]. These polymorphisms were not related to the therapeutic response to clozapine [50]. However, these polymorphisms are located outside the domain identified in our study. On the other hand, a polymorphism in the large intracellular domain region within the 5-HT_3A_ receptor gene has recently been shown to affect the clinical response to risperidone treatment [51]. Although the genetic findings available so far suggest a putative role of the 5-HT_3A_ receptor gene in the pathophysiology of schizophrenia and the response to antipsychotic treatment, no data are available on genetic variants within the extracellular domain of this receptor in schizophrenic patients. As such, based on our results further genetic studies should look more closely at the respective sequence responsible for clozapine affinity to the 5-HT_3A_ receptor identified in the present study by detailed fine mapping strategies. The therapeutic relevance of the 5-HT_3_ receptor in schizophrenia has recently been underlined by a placebo-controlled study with the 5-HT_3_ receptor antagonist ondansetron as an add-on medication to a stable dose of risperidone [52], which showed a positive effect of ondansetron on negative symptoms and cognitive impairment.

Taken together, these studies suggest that the 5-HT_3A_ receptor may contribute to the therapeutic efficacy of clozapine in schizophrenia and that the extracellular sequence close to the transmembrane domain TM1 within the 5-HT_3A_ receptor identified in the present study may play a role for the unique pharmacological profile of clozapine.
